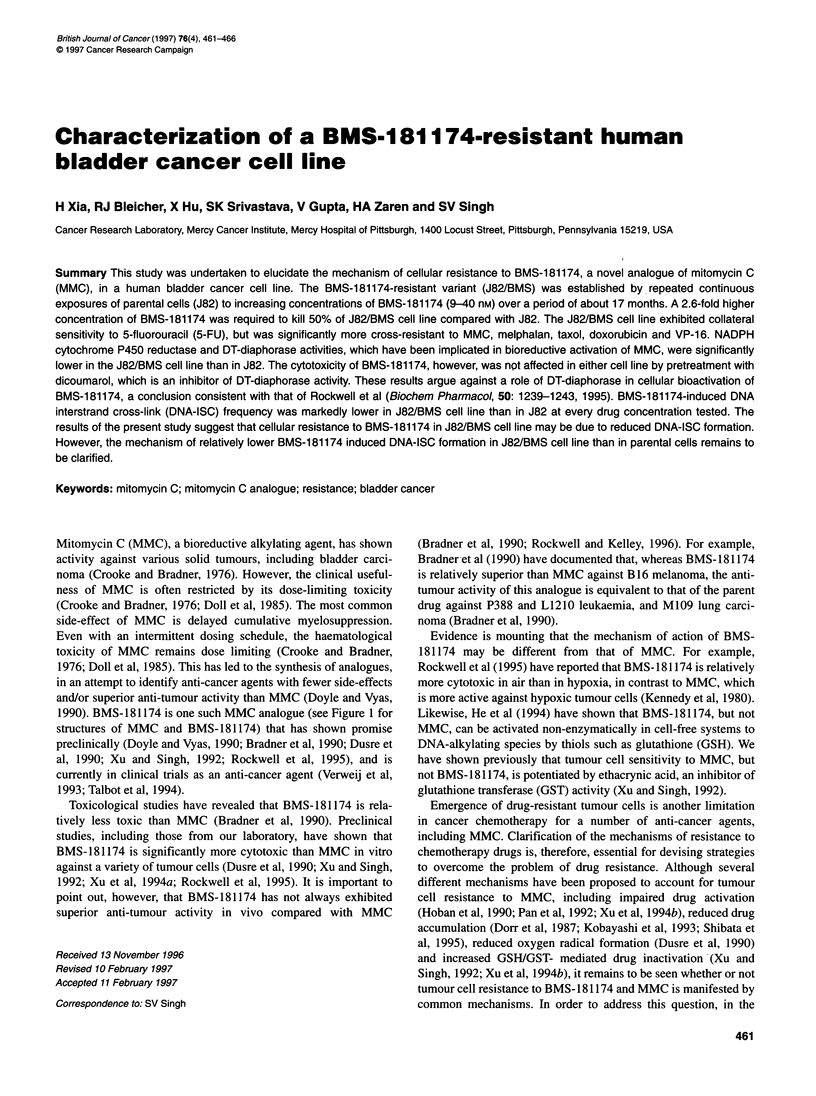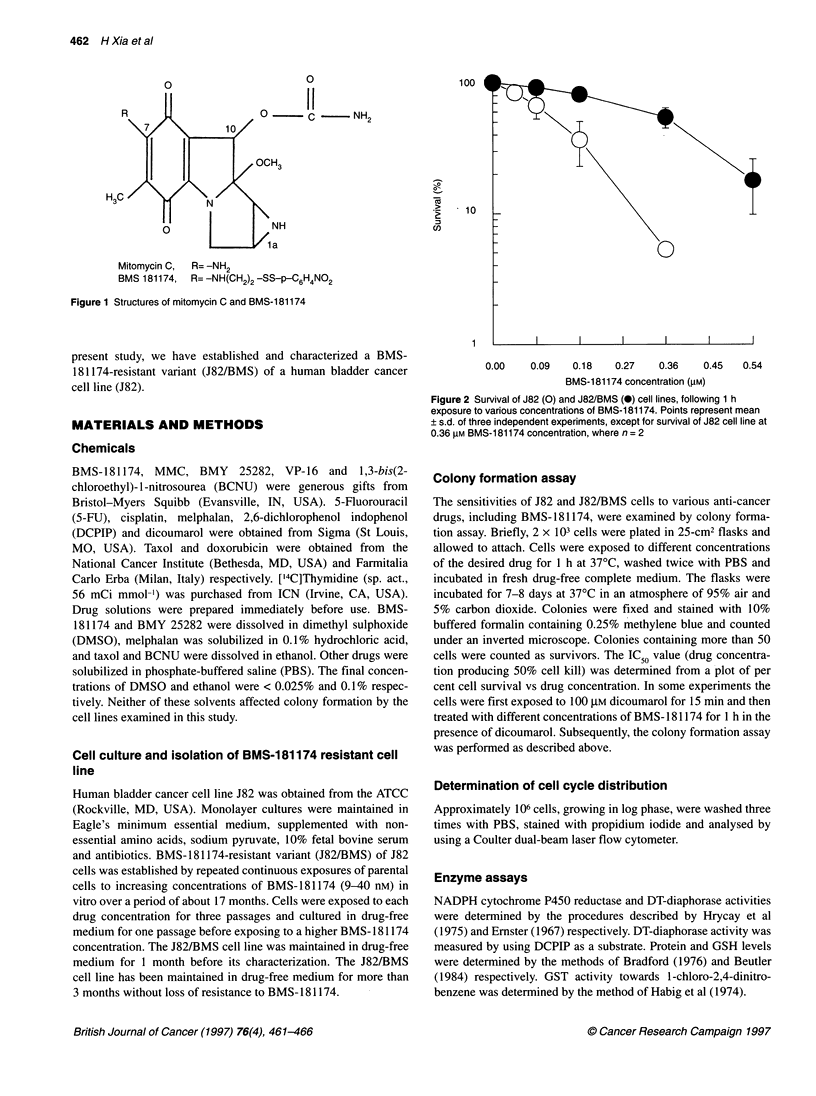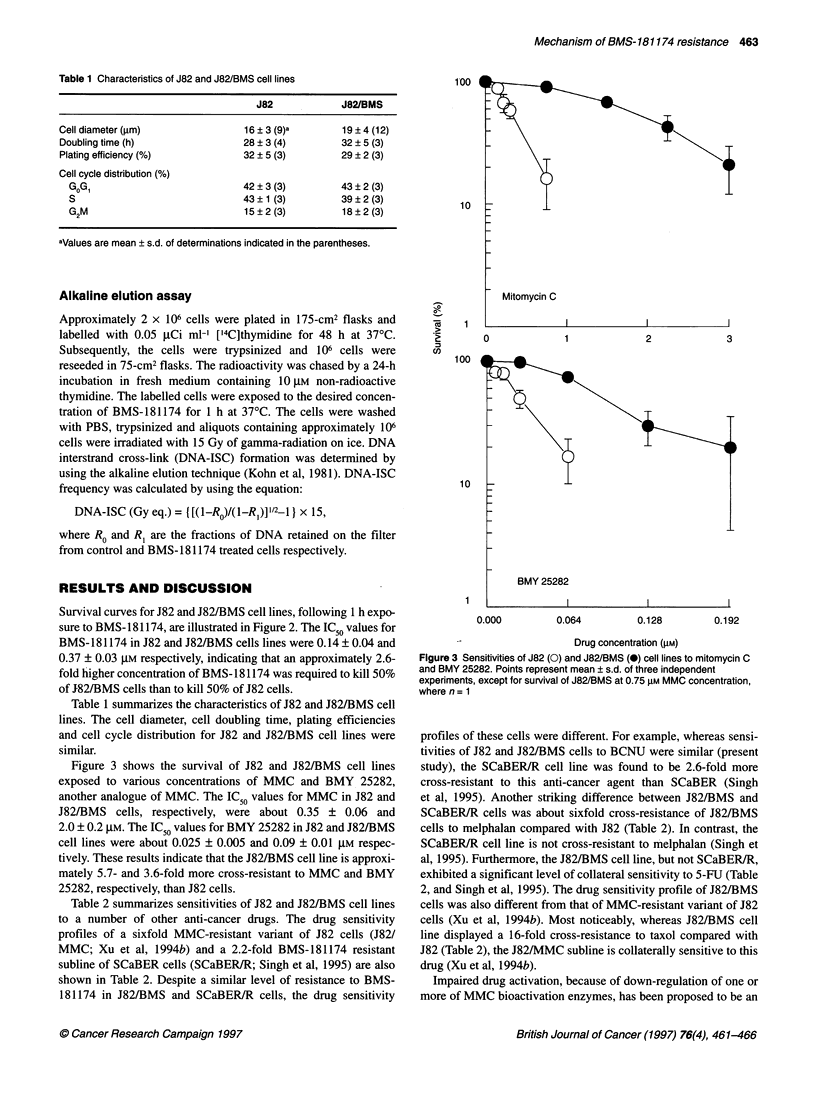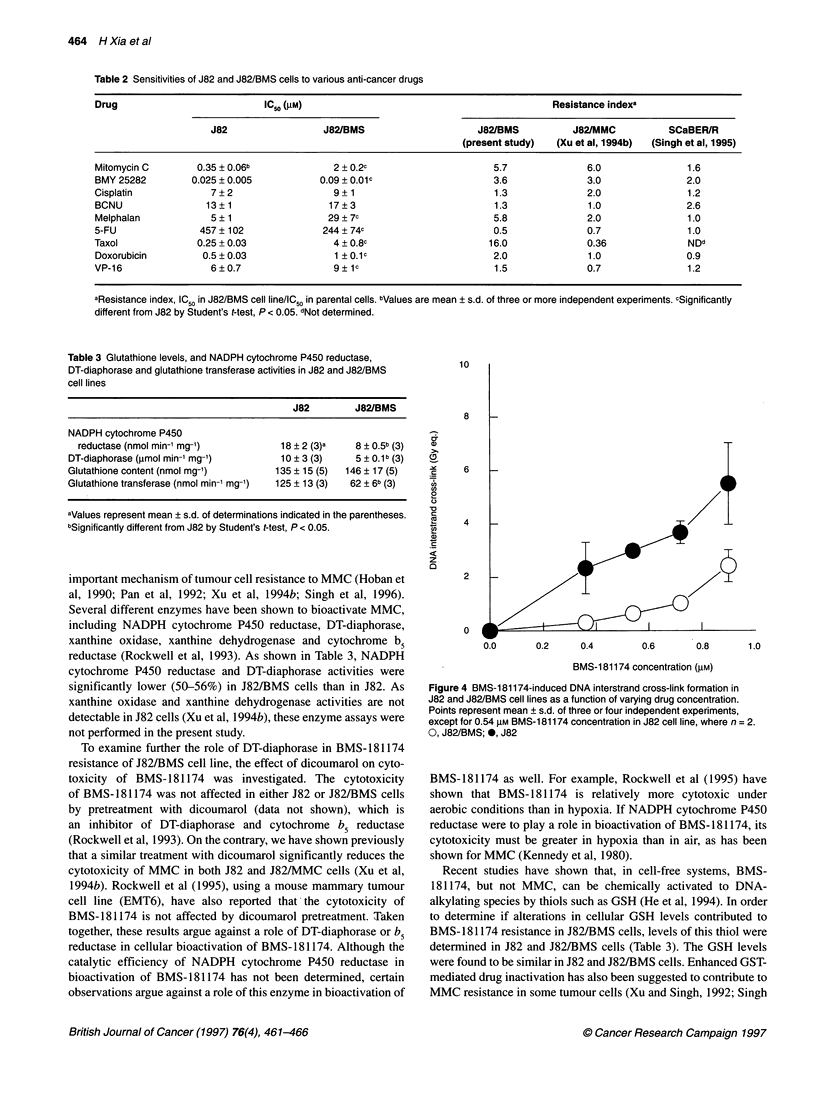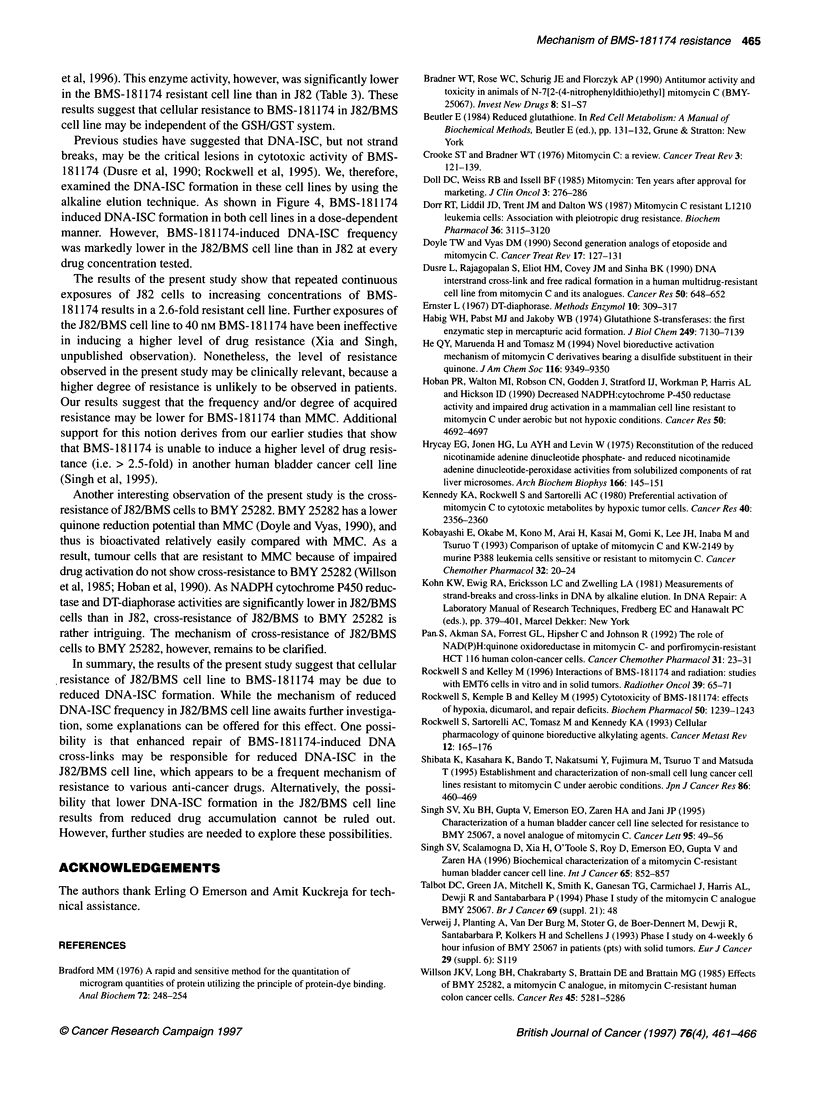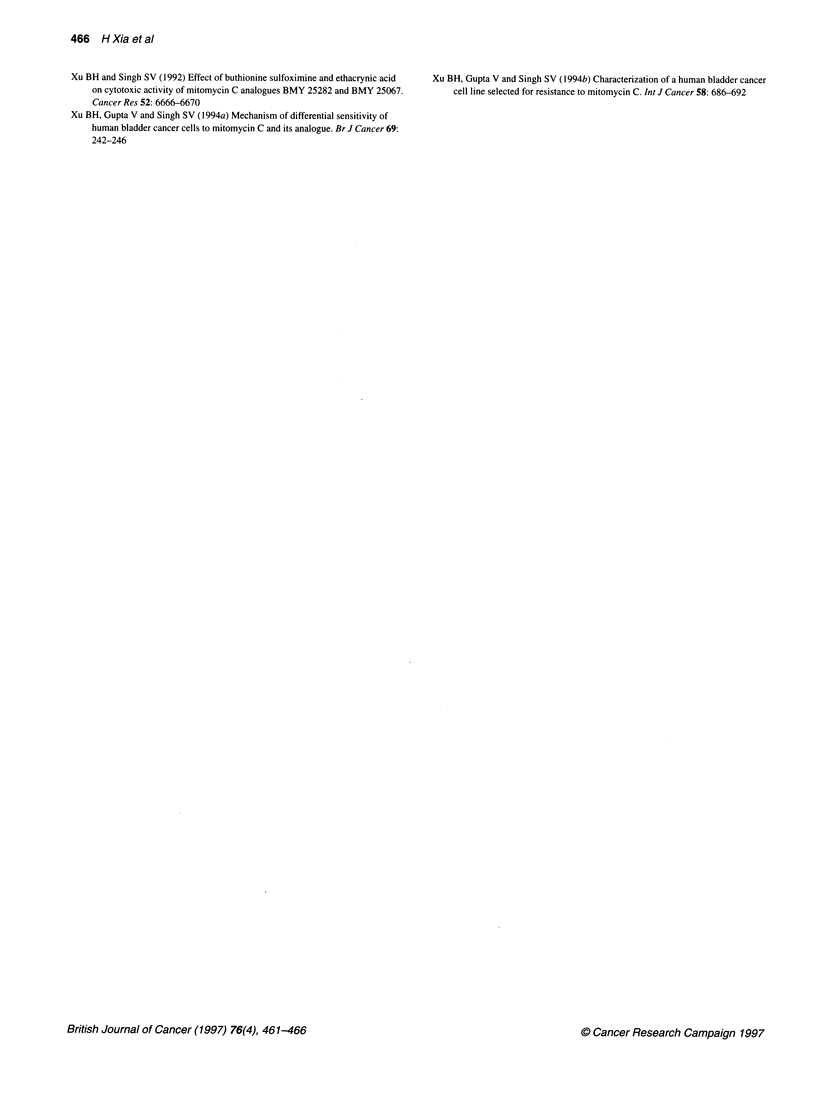# Characterization of a BMS-181174-resistant human bladder cancer cell line.

**DOI:** 10.1038/bjc.1997.410

**Published:** 1997

**Authors:** H. Xia, R. J. Bleicher, X. Hu, S. K. Srivastava, V. Gupta, H. A. Zaren, S. V. Singh

**Affiliations:** Cancer Research Laboratory, Mercy Cancer Institute, Mercy Hospital of Pittsburgh, Pennsylvania 15219, USA.

## Abstract

This study was undertaken to elucidate the mechanism of cellular resistance to BMS-181174, a novel analogue of mitomycin C (MMC), in a human bladder cancer cell line. The BMS-181174-resistant variant (J82/BMS) was established by repeated continuous exposures of parental cells (J82) to increasing concentrations of BMS-181174 (9-40 nM) over a period of about 17 months. A 2.6-fold higher concentration of BMS-181174 was required to kill 50% of J82/BMS cell line compared with J82. The J82/BMS cell line exhibited collateral sensitivity to 5-fluorouracil (5-FU), but was significantly more cross-resistant to MMC, melphalan, taxol, doxorubicin and VP-16. NADPH cytochrome P450 reductase and DT-diaphorase activities, which have been implicated in bioreductive activation of MMC, were significantly lower in the J82/BMS cell line than in J82. The cytotoxicity of BMS-181174, however, was not affected in either cell line by pretreatment with dicoumarol, which is an inhibitor of DT-diaphorase activity. These results argue against a role of DT-diaphorase in cellular bioactivation of BMS-181174, a conclusion consistent with that of Rockwell et al (Biochem Pharmacol, 50: 1239-1243, 1995). BMS-181174-induced DNA interstrand cross-link (DNA-ISC) frequency was markedly lower in J82/BMS cell line than in J82 at every drug concentration tested. The results of the present study suggest that cellular resistance to BMS-181174 in J82/BMS cell line may be due to reduced DNA-ISC formation. However, the mechanism of relatively lower BMS-181174 induced DNA-ISC formation in J82/BMS cell line than in parental cells remains to be clarified.


British Journal of Cancer (1997) 76(4), 461–466
© 1997 Cancer Research Campaign

### Characterization of a BMS-181174-resistant human bladder cancer cell line

**H Xia, RJ Bleicher, X Hu, SK Srivastava, V Gupta, HA Zaren and SV Singh**

Cancer Research Laboratory, Mercy Cancer Institute, Mercy Hospital of Pittsburgh, 1400 Locust Street, Pittsburgh, Pennsylvania 15219, USA

**Summary** This study was undertaken to elucidate the mechanism of cellular resistance to BMS-181174, a novel analogue of mitomycin C (MMC), in a human bladder cancer cell line. The BMS-181174-resistant variant (J82/BMS) was established by repeated continuous exposures of parental cells (J82) to increasing concentrations of BMS-181174 (9–40 nM) over a period of about 17 months. A 2.6-fold higher concentration of BMS-181174 was required to kill 50% of J82/BMS cell line compared with J82. The J82/BMS cell line exhibited collateral sensitivity to 5-fluorouracil (5-FU), but was significantly more cross-resistant to MMC, melphalan, taxol, doxorubicin and VP-16. NADPH cytochrome P450 reductase and DT-diaphorase activities, which have been implicated in bioreductive activation of MMC, were significantly lower in the J82/BMS cell line than in J82. The cytotoxicity of BMS-181174, however, was not affected in either cell line by pretreatment with dicoumarol, which is an inhibitor of DT-diaphorase activity. These results argue against a role of DT-diaphorase in cellular bioactivation of BMS-181174, a conclusion consistent with that of Rockwell et al (*Biochem Pharmacol*, **50**: 1239–1243, 1995). BMS-181174-induced DNA interstrand cross-link (DNA-ISC) frequency was markedly lower in J82/BMS cell line than in J82 at every drug concentration tested. The results of the present study suggest that cellular resistance to BMS-181174 in J82/BMS cell line may be due to reduced DNA-ISC formation. However, the mechanism of relatively lower BMS-181174 induced DNA-ISC formation in J82/BMS cell line than in parental cells remains to be clarified.

**Keywords:** mitomycin C; mitomycin C analogue; resistance; bladder cancer

Mitomycin C (MMC), a bioreductive alkylating agent, has shown activity against various solid tumours, including bladder carcinoma (Crooke and Bradner, 1976). However, the clinical usefulness of MMC is often restricted by its dose-limiting toxicity (Crooke and Bradner, 1976; Doll et al, 1985). The most common side-effect of MMC is delayed cumulative myelosuppression. Even with an intermittent dosing schedule, the haematological toxicity of MMC remains dose limiting (Crooke and Bradner, 1976; Doll et al, 1985). This has led to the synthesis of analogues, in an attempt to identify anti-cancer agents with fewer side-effects and/or superior anti-tumour activity than MMC (Doyle and Vyas, 1990). BMS-181174 is one such MMC analogue (see Figure 1 for structures of MMC and BMS-181174) that has shown promise preclinically (Doyle and Vyas, 1990; Bradner et al, 1990; Dusre et al, 1990; Xu and Singh, 1992; Rockwell et al, 1995), and is currently in clinical trials as an anti-cancer agent (Verweij et al, 1993; Talbot et al, 1994).

Toxicological studies have revealed that BMS-181174 is relatively less toxic than MMC (Bradner et al, 1990). Preclinical studies, including those from our laboratory, have shown that BMS-181174 is significantly more cytotoxic than MMC in vitro against a variety of tumour cells (Dusre et al, 1990; Xu and Singh, 1992; Xu et al, 1994*a*; Rockwell et al, 1995). It is important to point out, however, that BMS-181174 has not always exhibited superior anti-tumour activity in vivo compared with MMC (Bradner et al, 1990; Rockwell and Kelley, 1996). For example, Bradner et al (1990) have documented that, whereas BMS-181174 is relatively superior than MMC against B16 melanoma, the anti-tumour activity of this analogue is equivalent to that of the parent drug against P388 and L1210 leukaemia, and M109 lung carcinoma (Bradner et al, 1990).

Evidence is mounting that the mechanism of action of BMS-181174 may be different from that of MMC. For example, Rockwell et al (1995) have reported that BMS-181174 is relatively more cytotoxic in air than in hypoxia, in contrast to MMC, which is more active against hypoxic tumour cells (Kennedy et al, 1980). Likewise, He et al (1994) have shown that BMS-181174, but not MMC, can be activated non-enzymatically in cell-free systems to DNA-alkylating species by thiols such as glutathione (GSH). We have shown previously that tumour cell sensitivity to MMC, but not BMS-181174, is potentiated by ethacrynic acid, an inhibitor of glutathione transferase (GST) activity (Xu and Singh, 1992).

Emergence of drug-resistant tumour cells is another limitation in cancer chemotherapy for a number of anti-cancer agents, including MMC. Clarification of the mechanisms of resistance to chemotherapy drugs is, therefore, essential for devising strategies to overcome the problem of drug resistance. Although several different mechanisms have been proposed to account for tumour cell resistance to MMC, including impaired drug activation (Hoban et al, 1990; Pan et al, 1992; Xu et al, 1994*b*), reduced drug accumulation (Dorr et al, 1987; Kobayashi et al, 1993; Shibata et al, 1995), reduced oxygen radical formation (Dusre et al, 1990) and increased GSH/GST- mediated drug inactivation (Xu and Singh, 1992; Xu et al, 1994*b*), it remains to be seen whether or not tumour cell resistance to BMS-181174 and MMC is manifested by common mechanisms. In order to address this question, in the

*Received 13 November 1996*
*Revised 10 February 1997*
*Accepted 11 February 1997*

*Correspondence to:* SV Singh

**Figure 1** Structures of mitomycin C and BMS-181174

**Figure 2** Survival of J82 (O) and J82/BMS (●) cell lines, following 1 h exposure to various concentrations of BMS-181174. Points represent mean ± s.d. of three independent experiments, except for survival of J82 cell line at 0.36 μM BMS-181174 concentration, where $n = 2$

present study, we have established and characterized a BMS-181174-resistant variant (J82/BMS) of a human bladder cancer cell line (J82).

### MATERIALS AND METHODS

### Chemicals

BMS-181174, MMC, BMY 25282, VP-16 and 1,3-*bis*(2-chloroethyl)-1-nitrosourea (BCNU) were generous gifts from Bristol–Myers Squibb (Evansville, IN, USA). 5-Fluorouracil (5-FU), cisplatin, melphalan, 2,6-dichlorophenol indophenol (DCPIP) and dicoumarol were obtained from Sigma (St Louis, MO, USA). Taxol and doxorubicin were obtained from the National Cancer Institute (Bethesda, MD, USA) and Farmitalia Carlo Erba (Milan, Italy) respectively. [$^{14}$C]Thymidine (sp. act., 56 mCi mmol$^{-1}$) was purchased from ICN (Irvine, CA, USA). Drug solutions were prepared immediately before use. BMS-181174 and BMY 25282 were dissolved in dimethyl sulphoxide (DMSO), melphalan was solubilized in 0.1% hydrochloric acid, and taxol and BCNU were dissolved in ethanol. Other drugs were solubilized in phosphate-buffered saline (PBS). The final concentrations of DMSO and ethanol were < 0.025% and 0.1% respectively. Neither of these solvents affected colony formation by the cell lines examined in this study.

### Cell culture and isolation of BMS-181174 resistant cell line

Human bladder cancer cell line J82 was obtained from the ATCC (Rockville, MD, USA). Monolayer cultures were maintained in Eagle's minimum essential medium, supplemented with non-essential amino acids, sodium pyruvate, 10% fetal bovine serum and antibiotics. BMS-181174-resistant variant (J82/BMS) of J82 cells was established by repeated continuous exposures of parental cells to increasing concentrations of BMS-181174 (9–40 nM) in vitro over a period of about 17 months. Cells were exposed to each drug concentration for three passages and cultured in drug-free medium for one passage before exposing to a higher BMS-181174 concentration. The J82/BMS cell line was maintained in drug-free medium for 1 month before its characterization. The J82/BMS cell line has been maintained in drug-free medium for more than 3 months without loss of resistance to BMS-181174.

### Colony formation assay

The sensitivities of J82 and J82/BMS cells to various anti-cancer drugs, including BMS-181174, were examined by colony formation assay. Briefly, $2 \times 10^3$ cells were plated in 25-cm$^2$ flasks and allowed to attach. Cells were exposed to different concentrations of the desired drug for 1 h at 37°C, washed twice with PBS and incubated in fresh drug-free complete medium. The flasks were incubated for 7–8 days at 37°C in an atmosphere of 95% air and 5% carbon dioxide. Colonies were fixed and stained with 10% buffered formalin containing 0.25% methylene blue and counted under an inverted microscope. Colonies containing more than 50 cells were counted as survivors. The $IC_{50}$ value (drug concentration producing 50% cell kill) was determined from a plot of per cent cell survival vs drug concentration. In some experiments the cells were first exposed to 100 μM dicoumarol for 15 min and then treated with different concentrations of BMS-181174 for 1 h in the presence of dicoumarol. Subsequently, the colony formation assay was performed as described above.

### Determination of cell cycle distribution

Approximately $10^6$ cells, growing in log phase, were washed three times with PBS, stained with propidium iodide and analysed by using a Coulter dual-beam laser flow cytometer.

### Enzyme assays

NADPH cytochrome P450 reductase and DT-diaphorase activities were determined by the procedures described by Hrycay et al (1975) and Ernster (1967) respectively. DT-diaphorase activity was measured by using DCPIP as a substrate. Protein and GSH levels were determined by the methods of Bradford (1976) and Beutler (1984) respectively. GST activity towards 1-chloro-2,4-dinitrobenzene was determined by the method of Habig et al (1974).

 © Cancer Research Campaign 1997

**Table 1** Characteristics of J82 and J82/BMS cell lines

| | J82 | J82/BMS |
|---|---|---|
| Cell diameter (μm) | 16 ± 3 (9)[a] | 19 ± 4 (12) |
| Doubling time (h) | 28 ± 3 (4) | 32 ± 5 (3) |
| Plating efficiency (%) | 32 ± 5 (3) | 29 ± 2 (3) |
| Cell cycle distribution (%) | | |
| $G_0G_1$ | 42 ± 3 (3) | 43 ± 2 (3) |
| S | 43 ± 1 (3) | 39 ± 2 (3) |
| $G_2M$ | 15 ± 2 (3) | 18 ± 2 (3) |

[a]Values are mean ± s.d. of determinations indicated in the parentheses.

### Alkaline elution assay

Approximately 2 × $10^6$ cells were plated in 175-$cm^2$ flasks and labelled with 0.05 μCi $ml^{-1}$ [$^{14}C$]thymidine for 48 h at 37°C. Subsequently, the cells were trypsinized and $10^6$ cells were reseeded in 75-$cm^2$ flasks. The radioactivity was chased by a 24-h incubation in fresh medium containing 10 μM non-radioactive thymidine. The labelled cells were exposed to the desired concentration of BMS-181174 for 1 h at 37°C. The cells were washed with PBS, trypsinized and aliquots containing approximately $10^6$ cells were irradiated with 15 Gy of gamma-radiation on ice. DNA interstrand cross-link (DNA-ISC) formation was determined by using the alkaline elution technique (Kohn et al, 1981). DNA-ISC frequency was calculated by using the equation:

$$\text{DNA-ISC (Gy eq.)} = \{[(1-R_0)/(1-R_1)]^{1/2}-1\} \times 15,$$

where $R_0$ and $R_1$ are the fractions of DNA retained on the filter from control and BMS-181174 treated cells respectively.

### RESULTS AND DISCUSSION

Survival curves for J82 and J82/BMS cell lines, following 1 h exposure to BMS-181174, are illustrated in Figure 2. The $IC_{50}$ values for BMS-181174 in J82 and J82/BMS cells lines were 0.14 ± 0.04 and 0.37 ± 0.03 μM respectively, indicating that an approximately 2.6-fold higher concentration of BMS-181174 was required to kill 50% of J82/BMS cells than to kill 50% of J82 cells.

Table 1 summarizes the characteristics of J82 and J82/BMS cell lines. The cell diameter, cell doubling time, plating efficiencies and cell cycle distribution for J82 and J82/BMS cell lines were similar.

Figure 3 shows the survival of J82 and J82/BMS cell lines exposed to various concentrations of MMC and BMY 25282, another analogue of MMC. The $IC_{50}$ values for MMC in J82 and J82/BMS cells, respectively, were about 0.35 ± 0.06 and 2.0 ± 0.2 μM. The $IC_{50}$ values for BMY 25282 in J82 and J82/BMS cell lines were about 0.025 ± 0.005 and 0.09 ± 0.01 μM respectively. These results indicate that the J82/BMS cell line is approximately 5.7- and 3.6-fold more cross-resistant to MMC and BMY 25282, respectively, than J82 cells.

Table 2 summarizes sensitivities of J82 and J82/BMS cell lines to a number of other anti-cancer drugs. The drug sensitivity profiles of a sixfold MMC-resistant variant of J82 cells (J82/MMC; Xu et al, 1994*b*) and a 2.2-fold BMS-181174 resistant subline of SCaBER cells (SCaBER/R; Singh et al, 1995) are also shown in Table 2. Despite a similar level of resistance to BMS-181174 in J82/BMS and SCaBER/R cells, the drug sensitivity

**Figure 3** Sensitivities of J82 (○) and J82/BMS (●) cell lines to mitomycin C and BMY 25282. Points represent mean ± s.d. of three independent experiments, except for survival of J82/BMS at 0.75 μM MMC concentration, where $n = 1$

profiles of these cells were different. For example, whereas sensitivities of J82 and J82/BMS cells to BCNU were similar (present study), the SCaBER/R cell line was found to be 2.6-fold more cross-resistant to this anti-cancer agent than SCaBER (Singh et al, 1995). Another striking difference between J82/BMS and SCaBER/R cells was about sixfold cross-resistance of J82/BMS cells to melphalan compared with J82 (Table 2). In contrast, the SCaBER/R cell line is not cross-resistant to melphalan (Singh et al, 1995). Furthermore, the J82/BMS cell line, but not SCaBER/R, exhibited a significant level of collateral sensitivity to 5-FU (Table 2, and Singh et al, 1995). The drug sensitivity profile of J82/BMS cells was also different from that of MMC-resistant variant of J82 cells (Xu et al, 1994*b*). Most noticeably, whereas J82/BMS cell line displayed a 16-fold cross-resistance to taxol compared with J82 (Table 2), the J82/MMC subline is collaterally sensitive to this drug (Xu et al, 1994*b*).

Impaired drug activation, because of down-regulation of one or more of MMC bioactivation enzymes, has been proposed to be an

© Cancer Research Campaign 1997

**Table 2** Sensitivities of J82 and J82/BMS cells to various anti-cancer drugs

| Drug | $IC_{50}$ (μM) | | Resistance index[a] | | |
|---|---|---|---|---|---|
| | J82 | J82/BMS | J82/BMS (present study) | J82/MMC (Xu et al, 1994b) | SCaBER/R (Singh et al, 1995) |
| Mitomycin C | 0.35 ± 0.06[b] | 2 ± 0.2[c] | 5.7 | 6.0 | 1.6 |
| BMY 25282 | 0.025 ± 0.005 | 0.09 ± 0.01[c] | 3.6 | 3.0 | 2.0 |
| Cisplatin | 7 ± 2 | 9 ± 1 | 1.3 | 2.0 | 1.2 |
| BCNU | 13 ± 1 | 17 ± 3 | 1.3 | 1.0 | 2.6 |
| Melphalan | 5 ± 1 | 29 ± 7[c] | 5.8 | 2.0 | 1.0 |
| 5-FU | 457 ± 102 | 244 ± 74[c] | 0.5 | 0.7 | 1.0 |
| Taxol | 0.25 ± 0.03 | 4 ± 0.8[c] | 16.0 | 0.36 | ND[d] |
| Doxorubicin | 0.5 ± 0.03 | 1 ± 0.1[c] | 2.0 | 1.0 | 0.9 |
| VP-16 | 6 ± 0.7 | 9 ± 1[c] | 1.5 | 0.7 | 1.2 |

[a]Resistance index, $IC_{50}$ in J82/BMS cell line/$IC_{50}$ in parental cells. [b]Values are mean ± s.d. of three or more independent experiments. [c]Significantly different from J82 by Student's *t*-test, $P < 0.05$. [d]Not determined.

**Table 3** Glutathione levels, and NADPH cytochrome P450 reductase, DT-diaphorase and glutathione transferase activities in J82 and J82/BMS cell lines

| | J82 | J82/BMS |
|---|---|---|
| NADPH cytochrome P450 reductase (nmol $min^{-1}$ $mg^{-1}$) | 18 ± 2 (3)[a] | 8 ± 0.5[b] (3) |
| DT-diaphorase (μmol $min^{-1}$ $mg^{-1}$) | 10 ± 3 (3) | 5 ± 0.1[b] (3) |
| Glutathione content (nmol $mg^{-1}$) | 135 ± 15 (5) | 146 ± 17 (5) |
| Glutathione transferase (nmol $min^{-1}$ $mg^{-1}$) | 125 ± 13 (3) | 62 ± 6[b] (3) |

[a]Values represent mean ± s.d. of determinations indicated in the parentheses. [b]Significantly different from J82 by Student's *t*-test, $P < 0.05$.

important mechanism of tumour cell resistance to MMC (Hoban et al, 1990; Pan et al, 1992; Xu et al, 1994*b*; Singh et al, 1996). Several different enzymes have been shown to bioactivate MMC, including NADPH cytochrome P450 reductase, DT-diaphorase, xanthine oxidase, xanthine dehydrogenase and cytochrome $b_5$ reductase (Rockwell et al, 1993). As shown in Table 3, NADPH cytochrome P450 reductase and DT-diaphorase activities were significantly lower (50–56%) in J82/BMS cells than in J82. As xanthine oxidase and xanthine dehydrogenase activities are not detectable in J82 cells (Xu et al, 1994*b*), these enzyme assays were not performed in the present study.

To examine further the role of DT-diaphorase in BMS-181174 resistance of J82/BMS cell line, the effect of dicoumarol on cytotoxicity of BMS-181174 was investigated. The cytotoxicity of BMS-181174 was not affected in either J82 or J82/BMS cells by pretreatment with dicoumarol (data not shown), which is an inhibitor of DT-diaphorase and cytochrome $b_5$ reductase (Rockwell et al, 1993). On the contrary, we have shown previously that a similar treatment with dicoumarol significantly reduces the cytotoxicity of MMC in both J82 and J82/MMC cells (Xu et al, 1994*b*). Rockwell et al (1995), using a mouse mammary tumour cell line (EMT6), have also reported that the cytotoxicity of BMS-181174 is not affected by dicoumarol pretreatment. Taken together, these results argue against a role of DT-diaphorase or $b_5$ reductase in cellular bioactivation of BMS-181174. Although the catalytic efficiency of NADPH cytochrome P450 reductase in bioactivation of BMS-181174 has not been determined, certain observations argue against a role of this enzyme in bioactivation of

**Figure 4** BMS-181174-induced DNA interstrand cross-link formation in J82 and J82/BMS cell lines as a function of varying drug concentration. Points represent mean ± s.d. of three or four independent experiments, except for 0.54 μM BMS-181174 concentration in J82 cell line, where $n = 2$. ○, J82/BMS; ●, J82

BMS-181174 as well. For example, Rockwell et al (1995) have shown that BMS-181174 is relatively more cytotoxic under aerobic conditions than in hypoxia. If NADPH cytochrome P450 reductase were to play a role in bioactivation of BMS-181174, its cytotoxicity must be greater in hypoxia than in air, as has been shown for MMC (Kennedy et al, 1980).

Recent studies have shown that, in cell-free systems, BMS-181174, but not MMC, can be chemically activated to DNA-alkylating species by thiols such as GSH (He et al, 1994). In order to determine if alterations in cellular GSH levels contributed to BMS-181174 resistance in J82/BMS cells, levels of this thiol were determined in J82 and J82/BMS cells (Table 3). The GSH levels were found to be similar in J82 and J82/BMS cells. Enhanced GST-mediated drug inactivation has also been suggested to contribute to MMC resistance in some tumour cells (Xu and Singh, 1992; Singh

© Cancer Research Campaign 1997

et al, 1996). This enzyme activity, however, was significantly lower in the BMS-181174 resistant cell line than in J82 (Table 3). These results suggest that cellular resistance to BMS-181174 in J82/BMS cell line may be independent of the GSH/GST system.

Previous studies have suggested that DNA-ISC, but not strand breaks, may be the critical lesions in cytotoxic activity of BMS-181174 (Dusre et al, 1990; Rockwell et al, 1995). We, therefore, examined the DNA-ISC formation in these cell lines by using the alkaline elution technique. As shown in Figure 4, BMS-181174 induced DNA-ISC formation in both cell lines in a dose-dependent manner. However, BMS-181174-induced DNA-ISC frequency was markedly lower in the J82/BMS cell line than in J82 at every drug concentration tested.

The results of the present study show that repeated continuous exposures of J82 cells to increasing concentrations of BMS-181174 results in a 2.6-fold resistant cell line. Further exposures of the J82/BMS cell line to 40 nM BMS-181174 have been ineffective in inducing a higher level of drug resistance (Xia and Singh, unpublished observation). Nonetheless, the level of resistance observed in the present study may be clinically relevant, because a higher degree of resistance is unlikely to be observed in patients. Our results suggest that the frequency and/or degree of acquired resistance may be lower for BMS-181174 than MMC. Additional support for this notion derives from our earlier studies that show that BMS-181174 is unable to induce a higher level of drug resistance (i.e. > 2.5-fold) in another human bladder cancer cell line (Singh et al, 1995).

Another interesting observation of the present study is the cross-resistance of J82/BMS cells to BMY 25282. BMY 25282 has a lower quinone reduction potential than MMC (Doyle and Vyas, 1990), and thus is bioactivated relatively easily compared with MMC. As a result, tumour cells that are resistant to MMC because of impaired drug activation do not show cross-resistance to BMY 25282 (Willson et al, 1985; Hoban et al, 1990). As NADPH cytochrome P450 reductase and DT-diaphorase activities are significantly lower in J82/BMS cells than in J82, cross-resistance of J82/BMS to BMY 25282 is rather intriguing. The mechanism of cross-resistance of J82/BMS cells to BMY 25282, however, remains to be clarified.

In summary, the results of the present study suggest that cellular resistance of J82/BMS cell line to BMS-181174 may be due to reduced DNA-ISC formation. While the mechanism of reduced DNA-ISC frequency in J82/BMS cell line awaits further investigation, some explanations can be offered for this effect. One possibility is that enhanced repair of BMS-181174-induced DNA cross-links may be responsible for reduced DNA-ISC in the J82/BMS cell line, which appears to be a frequent mechanism of resistance to various anti-cancer drugs. Alternatively, the possibility that lower DNA-ISC formation in the J82/BMS cell line results from reduced drug accumulation cannot be ruled out. However, further studies are needed to explore these possibilities.

### ACKNOWLEDGEMENTS

The authors thank Erling O Emerson and Amit Kuckreja for technical assistance.

### REFERENCES

Bradford MM (1976) A rapid and sensitive method for the quantitation of microgram quantities of protein utilizing the principle of protein-dye binding. *Anal Biochem* **72**: 248–254

Bradner WT, Rose WC, Schurig JE and Florczyk AP (1990) Antitumor activity and toxicity in animals of N-7[2-(4-nitrophenyldithio)ethyl] mitomycin C (BMY-25067). *Invest New Drugs* **8**: S1–S7

Beutler E (1984) Reduced glutathione. In *Red Cell Metabolism: A Manual of Biochemical Methods,* Beutler E (ed.), pp. 131–132, Grune & Stratton: New York

Crooke ST and Bradner WT (1976) Mitomycin C: a review. *Cancer Treat Rev* **3**: 121–139.

Doll DC, Weiss RB and Issell BF (1985) Mitomycin: Ten years after approval for marketing. *J Clin Oncol* **3**: 276–286

Dorr RT, Liddil JD, Trent JM and Dalton WS (1987) Mitomycin C resistant L1210 leukemia cells: Association with pleiotropic drug resistance. *Biochem Pharmacol* **36**: 3115–3120

Doyle TW and Vyas DM (1990) Second generation analogs of etoposide and mitomycin C. *Cancer Treat Rev* **17**: 127–131

Dusre L, Rajagopalan S, Eliot HM, Covey JM and Sinha BK (1990) DNA interstrand cross-link and free radical formation in a human multidrug-resistant cell line from mitomycin C and its analogues. *Cancer Res* **50**: 648–652

Ernster L (1967) DT-diaphorase. *Methods Enzymol* **10**: 309–317

Habig WH, Pabst MJ and Jakoby WB (1974) Glutathione S-transferases: the first enzymatic step in mercapturic acid formation. *J Biol Chem* **249**: 7130–7139

He QY, Maruenda H and Tomasz M (1994) Novel bioreductive activation mechanism of mitomycin C derivatives bearing a disulfide substituent in their quinone. *J Am Chem Soc* **116**: 9349–9350

Hoban PR, Walton MI, Robson CN, Godden J, Stratford IJ, Workman P, Harris AL and Hickson ID (1990) Decreased NADPH:cytochrome P-450 reductase activity and impaired drug activation in a mammalian cell line resistant to mitomycin C under aerobic but not hypoxic conditions. *Cancer Res* **50**: 4692–4697

Hrycay EG, Jonen HG, Lu AYH and Levin W (1975) Reconstitution of the reduced nicotinamide adenine dinucleotide phosphate- and reduced nicotinamide adenine dinucleotide-peroxidase activities from solubilized components of rat liver microsomes. *Arch Biochem Biophys* **166**: 145–151

Kennedy KA, Rockwell S and Sartorelli AC (1980) Preferential activation of mitomycin C to cytotoxic metabolites by hypoxic tumor cells. *Cancer Res* **40**: 2356–2360

Kobayashi E, Okabe M, Kono M, Arai H, Kasai M, Gomi K, Lee JH, Inaba M and Tsuruo T (1993) Comparison of uptake of mitomycin C and KW-2149 by murine P388 leukemia cells sensitive or resistant to mitomycin C. *Cancer Chemother Pharmacol* **32**: 20–24

Kohn KW, Ewig RA, Ericksson LC and Zwelling LA (1981) Measurements of strand-breaks and cross-links in DNA by alkaline elution. In DNA Repair: A Laboratory Manual of Research Techniques, Fredberg EC and Hanawalt PC (eds.), pp. 379–401, Marcel Dekker: New York

Pan S, Akman SA, Forrest GL, Hipsher C and Johnson R (1992) The role of NAD(P)H:quinone oxidoreductase in mitomycin C- and porfiromycin-resistant HCT 116 human colon-cancer cells. *Cancer Chemother Pharmacol* **31**: 23–31

Rockwell S and Kelley M (1996) Interactions of BMS-181174 and radiation: studies with EMT6 cells in vitro and in solid tumors. *Radiother Oncol* **39**: 65–71

Rockwell S, Kemple B and Kelley M (1995) Cytotoxicity of BMS-181174: effects of hypoxia, dicumarol, and repair deficits. *Biochem Pharmacol* **50**: 1239–1243

Rockwell S, Sartorelli AC, Tomasz M and Kennedy KA (1993) Cellular pharmacology of quinone bioreductive alkylating agents. *Cancer Metast Rev* **12**: 165–176

Shibata K, Kasahara K, Bando T, Nakatsumi Y, Fujimura M, Tsuruo T and Matsuda T (1995) Establishment and characterization of non-small cell lung cancer cell lines resistant to mitomycin C under aerobic conditions. *Jpn J Cancer Res* **86**: 460–469

Singh SV, Xu BH, Gupta V, Emerson EO, Zaren HA and Jani JP (1995) Characterization of a human bladder cancer cell line selected for resistance to BMY 25067, a novel analogue of mitomycin C. *Cancer Lett* **95**: 49–56

Singh SV, Scalamogna D, Xia H, O'Toole S, Roy D, Emerson EO, Gupta V and Zaren HA (1996) Biochemical characterization of a mitomycin C-resistant human bladder cancer cell line. *Int J Cancer* **65**: 852–857

Talbot DC, Green JA, Mitchell K, Smith K, Ganesan TG, Carmichael J, Harris AL, Dewji R and Santabarbara P (1994) Phase I study of the mitomycin C analogue BMY 25067. *Br J Cancer* **69** (suppl. 21): 48

Verweij J, Planting A, Van Der Burg M, Stoter G, de Boer-Dennert M, Dewji R, Santabarbara P, Kolkers H and Schellens J (1993) Phase I study on 4-weekly 6 hour infusion of BMY 25067 in patients (pts) with solid tumors. *Eur J Cancer* **29** (suppl. 6): S119

Willson JKV, Long BH, Chakrabarty S, Brattain DE and Brattain MG (1985) Effects of BMY 25282, a mitomycin C analogue, in mitomycin C-resistant human colon cancer cells. *Cancer Res* **45**: 5281–5286

© Cancer Research Campaign 1997

Xu BH and Singh SV (1992) Effect of buthionine sulfoximine and ethacrynic acid on cytotoxic activity of mitomycin C analogues BMY 25282 and BMY 25067. *Cancer Res* **52**: 6666–6670

Xu BH, Gupta V and Singh SV (1994*a*) Mechanism of differential sensitivity of human bladder cancer cells to mitomycin C and its analogue. *Br J Cancer* **69**: 242–246

Xu BH, Gupta V and Singh SV (1994*b*) Characterization of a human bladder cancer cell line selected for resistance to mitomycin C. *Int J Cancer* **58**: 686–692

© Cancer Research Campaign 1997